# Effects of Wind Conditions on Wind Turbine Temperature Monitoring and Solution Based on Wind Condition Clustering and IGA-ELM

**DOI:** 10.3390/s22041516

**Published:** 2022-02-15

**Authors:** Zhengnan Hou, Shengxian Zhuang

**Affiliations:** 1 School of Electrical Engineering, Southwest Jiaotong University, Chengdu 610031, China; zhuangshengxian@swjtu-leeds.com.cn; 2 School of Electrical Engineering, Southwest Jiaotong University–University of Leeds Joint School, Chengdu 610031, China

**Keywords:** wind turbine, temperature monitoring, wind condition clustering, IGA-ELM, SCADA

## Abstract

To reduce maintenance costs of wind turbines (WTs), WT health monitoring has attracted wide attention, and different methods have been proposed. However, most existing WT temperature monitoring methods ignore the fact that various wind conditions can directly affect internal temperature of WT, such as main bearing temperature. This paper analyzes the effects of wind conditions on WT temperature monitoring. To reduce these effects, this paper also proposes a novel WT temperature monitoring solution. Compared with existing solutions, the proposed solution has two advantages: (1) wind condition clustering (WCC) is applied and then a normal turbine behavior model is built for each wind condition; (2) extreme learning machine (ELM) is optimized by an improved genetic algorithm (IGA) to avoid local minimum due to the irregularity of wind condition change and the randomness of initial coefficients. Cases of real SCADA data validate the effectiveness and advantages of the proposed solution.

## 1. Introduction

Since wind energy is renewable and pollution-free, many governments cite wind energy as a primary future energy source [1]. However, the high maintenance costs of wind turbines (WTs) seriously restrict the development of the wind energy industry [2]. The most effective way to reduce maintenance costs is to monitor the working state of WTs to sound alarms when failures occur. Thus, model-based WT monitoring has attracted wide attention, and different methods have been proposed. Existing model-based WT monitoring methods can be roughly divided into two categories [3]: theoretical and data-driven. The advantage of theoretical methods is that fewer data are required. By using theoretical methods, main physical mechanisms can be responsible for the temperature changes [4]; annual energy is obtained [5]. For data-driven methods, model accuracy relies on the quality and quantity of the data. By using data-driven methods, turbines with weakened power generation performance are identified through assessing the wind power curve profiles [6]; short-horizon power is predicted [7]; maintenance decisions are made according to the complex WT component degradation processes [8]; fault can be detected [9]. Since WTs are complex electromechanical systems, the relationships between its various parameters are primarily nonlinear; thus, the construction of the theoretical model is difficult and inaccurate [10]. With supervisory control and data acquisition (SCADA) improvements [11,12,13], the data-driven methods are more suitable for WT monitoring [14].

A typical data-driven WT monitoring method analyzes real-time data using a model with one parameter as the output and other parameters as inputs [15]. When building data-driven models, several different intelligent algorithms are applied. With nonlinear state estimate technology (NSET), the tower vibration model [16] and gearbox oil temperature model [17] are built. With logistic regression (LR), direct-drive wind power generation set [18] and bearing performance condition [19] are analyzed. With the support vector machine (SVM), a WT bearing model is built [19]; diagonal spectrum and a clustering binary tree are obtained [20]; a multi-sensory system [21] and a two-stage fault detection scheme [22] for fault diagnosis is presented. In addition to the above algorithms, neural network (NN) is widely applied due to its high accuracy and short training time. With NN, a robust simultaneous estimate of actuator faults and system states is achieved [23]; wavelet transformed power components open-circuit faults are identified [24]; dynamic equations modeling wind power output, vibration of drive train and vibration of tower are captured [25]. Since SCADA of wind farm can obtain very large datasets, NN would need heavy computational costs [26]. Same as NN, extreme learning machine (ELM) can assess prediction intervals of time-series predictions [27]. Different from NN, the input weights and hidden layer biases in extreme learning machine (ELM) are assigned randomly, and the output weights of the hidden layer are directly calculated by a Moore–Penrose (MP) generalized inverse operation [28,29,30]. Consequently, ELM is more computationally efficient than NN [31].

Although the existing methods are based on several different algorithms, the main ideas are basically same, which are to construct accurate WT normal behavior models. However, most existing methods ignore the variable wind conditions which can directly affect WT internal parameters. For the methods considering wind conditions, the wind condition clustering (WCC) is primarily based on the absolute value of the wind speed. This type of WCC is effective for WT power monitoring but not accurate for WT temperature monitoring, since wind condition is directly related to internal temperature parameters [32,33]. Through research on SCADA data, the internal temperature is found to exhibit certain differences during different wind conditions. For example, the temperature difference of one same main bearing between wind speed increase and decrease may be more than 5 °C under the same wind speed of 10 m/s. If WCC only depends on the absolute value of wind speed, the temperature monitoring result may be inaccurate. In addition, wind condition changes are irregular, which can mislead the intelligent algorithm of a data-driven method. For example, the wind speed of more than 12.5 m/s can keep for 1 day. If this period is in the learning set, the intelligent algorithm may determine that the wind speed has little effect on the WT’s internal temperature. Conversely, the wind speed can also rise from 0 to 15 m/s in 2 h, which may cause the WT’s internal temperature to change a lot. If the learning set includes one (or more) periods like that, the intelligent algorithm may determine that the wind speed has a strong impact on the WT’s internal temperature. Both situations arise due to the irregularity of wind condition.

To address these issues, a novel WT temperature monitoring solution is proposed in this paper. Considering the effects of wind condition on the WT’s internal temperature, a WCC scheme using k-means algorithm is proposed. This is a divide-and-conquer strategy and can enable the building of models in different wind conditions, which can improve the reliability and accuracy of fault detection. The proposed solution also uses an improved genetic algorithm (IGA) to optimize ELM to avoid local minimum due to the irregularity of wind condition change and the randomness of initial weights and bias.

In general, the main innovations of this paper are as follows:

1. The effects of wind condition on a WT’s internal temperature are investigated.
2. A WCC scheme is proposed so that normal WT behaviors are built under different clusters. This divide-and-conquer strategy can help reduce false alarms.
3. IGA is used to optimize ELM to improve the accuracy of the model.

The remainder of this paper is organized as follows. Section 2 investigates the effects of wind conditions on WT temperature monitoring. In Section 3, the framework of the proposed solution is presented in detail. Section 4 presents the cases study and the results of various analyses. Conclusions are summarized in Section 5.

## 2. Effects of Wind Conditions

**Figure 1:**
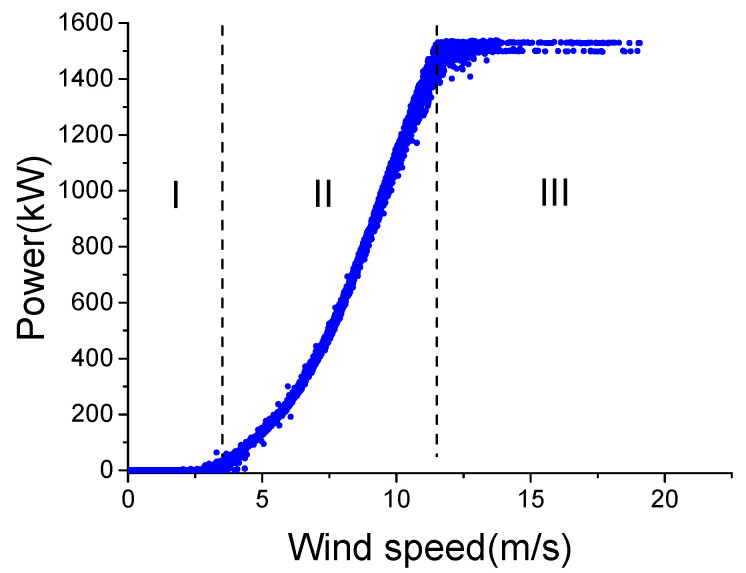
Active power-wind speed in normal state.

**Figure 2:**
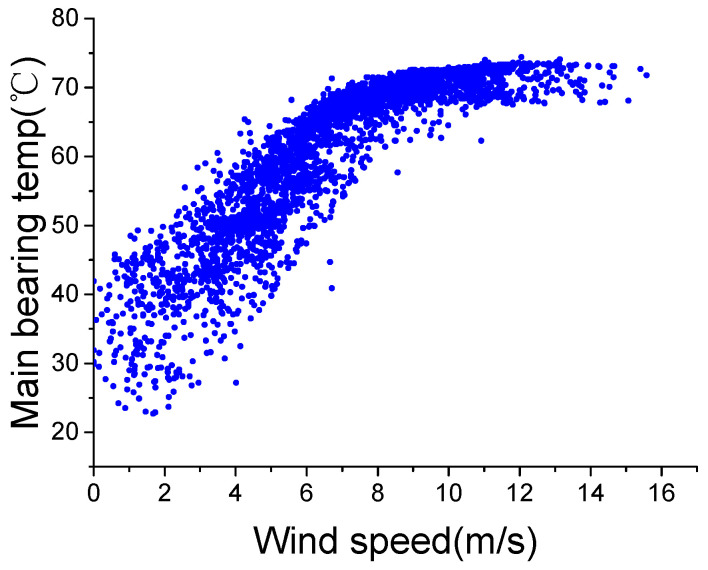
Main bearing temperature-wind speed in normal state.

WTs are exposed to harsh weather conditions all year round. Large differences between wind conditions can make the internal temperatures of different components vary markedly, even under the same working state. Figure 1 shows the active power-wind speed curve under a normal working state, and Figure 2 shows the main bearing temperature-wind speed curve. To reduce the impact of external temperature, the external temperature of the two data sets shown in the two curves is between 14 and 16 °C. 

The two figures show that the active power and internal temperature are directly related to the wind speed, which means WT temperature monitoring should consider wind speed. Therefore, most existing WCC divide wind conditions into three regions: I (0–3 m/s), II (3–12 m/s) and III (>12 m/s) according to the absolute value of wind speed, as shown in Figure 1, without considering wind speed change. However, the fact is that wind speed change also affects internal temperatures.

**Figure 3:**
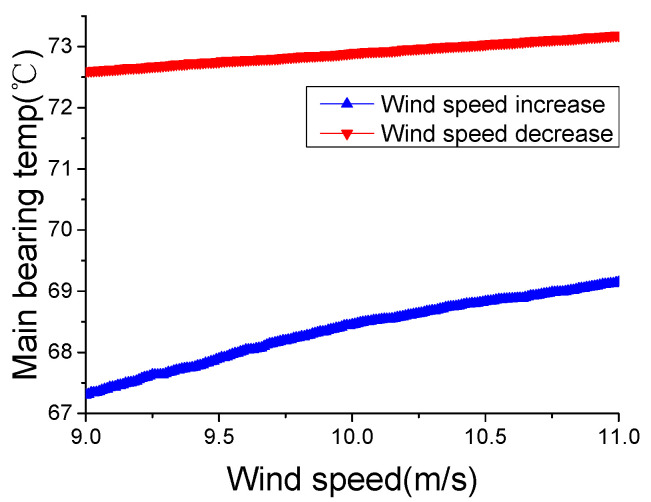
Main bearing temperature during a wind speed increase and decrease.

Figure 2 shows that the main bearing temperature increases as the wind speed increases; this result occurs because wind speed has a direct positive correlation with rotor speed, which is directly related to the heat generated in a WT. Considering the progress of the heat conduction, there can be some delay between the wind speed change and internal temperature change. Due to this delay, under the same wind speed, the main bearing temperature during an increase in wind speed could be lower than during a decrease in wind speed. In order to present this phenomenon specifically, the main bearing temperature-wind speed curves during wind speed increase and decrease are shown in Figure 3. For an accurate description, despite the wind speed change, the working state and external temperature are similar, which are producing an active power of 900–1000 kW, an external temperature of 14–16 °C and a wind speed of 9–11 m/s.

Figure 3 shows that, under the same wind speed, the main bearing temperature experiences a significant difference during wind speed increase and decrease. The average difference is 4.6 °C, and the maximum difference can reach 5.4 °C. These results indicate that changes of wind speed affect internal temperatures in a WT. Thus, WCC should consider not only the absolute value of wind speed but also the changes of that.

More importantly, wind condition is particularly irregular that wind speed can be constant for a long time or fluctuate markedly in a short time, making intelligent algorithms arrive at a local minimum. Therefore, to improve the accuracy of WT temperature monitoring, it is necessary to optimize the intelligent algorithm for the solution with WCC.

## 3. Proposed Solution Framework

**Figure 4:**
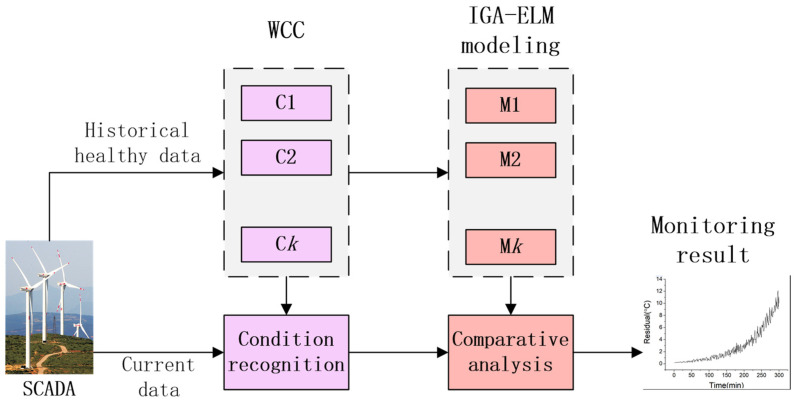
Flowchart of the proposed solution with WCC and IGA-ELM using SCADA.

To reduce the effects of wind conditions and monitoring WT temperature accurately, a solution with WCC and IGA-ELM is proposed, and its flowchart is shown in Figure 4. The proposed solution has two key parts:

- Wind data are partitioned into several condition clusters by using *K*-means clustering, so that each wind condition has an independent normal behavior model. This can make the monitored data more suitable with their corresponding models. To our best knowledge, this is the first WCC based on a data-driven method.
- The ELM algorithm is based on one set of initial input weights and hidden layer bias, which could cause the ELM models fail to achieve its due accuracy. In the proposed solution, IGA, with the random global search capability, is applied to optimize ELM for the irregularity of wind condition change and the randomness of initial weights and bias.

With historical healthy data, WCC and IGA-ELM models can simulate the normal behaviors of WT. When monitoring, the residuals, which are the difference between the actual value and the predicted value of the models, are analyzed to show the deviation of the actual state from the normal behaviors. The rise of the residuals can be an indication of possible failure.

### 3.1. WCC Using K-Means Clustering

In the proposed solution, *K*-means clustering is applied for WCC. As an unsupervised learning method, *K*-means clustering is widely used in clustering algorithms due to its accuracy and efficiency [34]. The aim of *K*-means clustering is to allocate all wind condition samples into *K* clusters by minimizing the sum of the squared error over all *K* clusters, denoted as follows [35]:

(1) $$d_{i}=\arg\underset{O}{\min}\underset{x_{i}\in O_{i}}{\sum }{\left||l_{i}-\mu_{i}|\right|}^{2}$$

where $O=\left\{O_{1},O_{2},\ldots ,O_{k}\right\}$ is the set of *K* clusters, $\mu_{i}$ is the cluster centroid of the *i*th cluster, $\left\{l_{1},l_{2},\ldots ,l_{N}\right\}$ is the cluster samples, and *N* is the number of samples.

In the *K*-means algorithm, the clustering number *K* is a key parameter. To evaluate the clustering number *K*, the silhouette value [36] is applied in the proposed solution. The silhouette value for the *i*th sample is expressed as

(2) $$S_{i}=\frac{b_{i}-a_{i}}{\max\left\{a_{i},b_{i}\right\}}$$

where $a_{i}=\frac{\sum_{x^{\prime }\in O_{i},x\neq x^{\prime }}dist\left(x,x^{\prime }\right)}{\left|O_{i}\right|-1}$ is the average distance from the *i*th sample to other samples from the same cluster and $b_{i}=\underset{j\neq i}{\min}\left\{\frac{\sum_{x^{\prime }\in O_{j}}dist\left(x,x^{\prime }\right)}{\left|O_{j}\right|}\right\}$ is the minimum average distance from the *i*th sample to other samples from different clusters. The range of $S_{i}$ is [−1, 1]. $S_{i}$ indicates the rationality of the *i*th sample’s clustering, and the average of all $S_{i}$ indicates the rationality of WCC with *K* clusters.

### 3.2. WT Model Based on IGA-ELM

#### 3.2.1. ELM Algorithm

**Figure 5:**
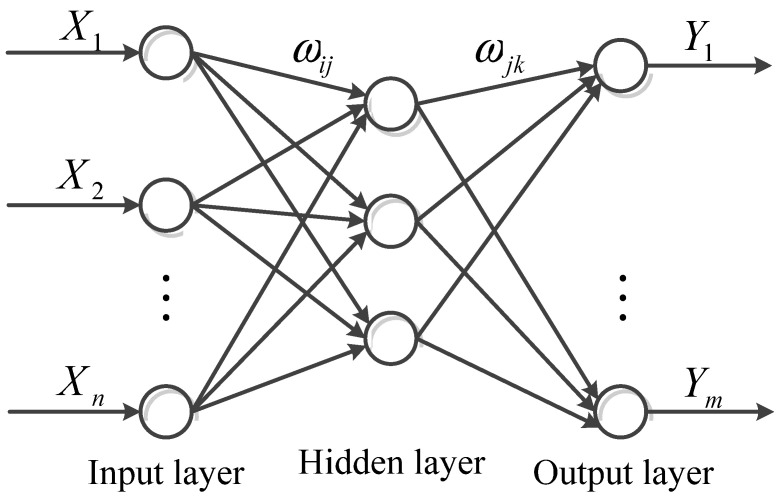
Topology of the extreme learning machine (ELM).

The intelligent algorithm is the key to the model-based WT temperature monitoring. Compared with NN, the ELM has the advantages of fast training speed and high accuracy. ELM is composed of a single hidden layer feed forward neural network. The topological diagram of ELM is shown as in Figure 5.

In Figure 4, $X={\left[X_{1},X_{2},\ldots ,X_{n}\right]}^{T}\in R^{n}$ and $Y={\left[Y_{1},Y_{2},\ldots ,Y_{m}\right]}^{T}\in R^{m}$ are the inputs and outputs of the model, respectively; $\omega_{ij}$ and $\omega_{jk}$ is the input and output weights, respectively. For *n* distinct samples ***X***, the ELM can approximate the target as

(3) $$\hat{Y_{k}}=\sum_{i=1}^{m}\overset{\tilde{n}}{\underset{j=1}{\sum }}\omega_{jk}\cdot g\left(\omega_{ij}\cdot X_{i}+b_{i}\right)$$

where g(·) represents the activation function, $\tilde{n}$ is the number of hidden nodes, and $b_{i}$ is the hidden layer bias.

If ELM can fit *n* distinct samples with zero error, the matrix form of approximation can be expressed as

(4) $$Y=\hat{Y}=H\omega_{\tilde{n}m}$$

where the output weights $\omega_{\tilde{n}m}={\left[\omega_{1}^{T},\omega_{2}^{T},\ldots ,\omega_{m}^{T}\right]}^{T}$, $\omega_{k}={\left[\omega_{1k},\omega_{2k},\ldots ,\omega_{\tilde{n}k}\right]}^{T}$ and the hidden layer output matrix H can be expressed as

(5) $$H={\left[\begin{array}{ccc} g\left(\omega_{11}\cdot X_{1}+b_{1}\right) & \ldots & g\left(\omega_{\tilde{n}1}\cdot X_{1}+b_{\tilde{n}}\right)\\ \ldots & \ldots & \ldots \\ g\left(\omega_{1m}\cdot X_{m}+b_{1}\right) & \ldots & g\left(\omega_{\tilde{n}m}\cdot X_{m}+b_{\tilde{n}}\right) \end{array}\right]}_{m\times \tilde{n}}$$

With given input weights $\omega_{ij}$ and hidden layer bias $b_{j}$, the output weight can be analytically calculated by a least squares method as

(6) $$\left||H{\hat{\omega }}_{\tilde{n}m}-Y||=||{HH}^{+}Y-Y||=\underset{\omega_{\tilde{n}m}}{\min}||H\omega_{\tilde{n}m}-Y|\right|$$

where $H^{+}$ is the generalized Moore Penrose inverse of H.

Then the solution can be expressed as

(7) $${\hat{\omega }}_{\tilde{n}n}=H^{+}Y$$

#### 3.2.2. GA Optimization

The ELM model is based on one set of initial input weights $\omega_{ij}$ and hidden layer bias $b_{j}$, which are set based on experience in most existing studies [28,29,30]. However, the wind conditions are irregular. This makes the ELM model, with initial coefficients based on experience, fail to achieve its due accuracy. To solve this problem, GA is applied to optimize ELM in the proposed solution. GA is a global random search optimization algorithm based on the genetic mechanism and evolution, which can select the individuals with good fitness. With the strong global search capability, the initial coefficients of ELM can be optimized by GA and the accuracy of the model can be improved [37,38].

The coefficients to be optimized, which are initial input weights $\omega_{ij}$ and hidden layer bias $b_{j}$ of ELM in this study, are coded as individual chromosome. The fitness *F*, which can judge whether the code is a good solution, is calculated as

(8) $$F=1/\left(\overset{m}{\underset{i=1}{\sum }}\left|e_{k}\right|\right)$$

where $e_{k}$ is error of ELM as $e_{k}=Y_{k}-\hat{Y_{k}}$ and *m* is the number of output layer nodes.

The GA optimization process proceeds as follows:

- Step 1, selection. GA selection is based on fitness, and the probability of selection is calculated as

(9) $$p_{i}=\frac{k/F_{i}}{\sum_{j=1}^{N}k/F_{i}}$$

where *N* is the number of individuals;

- Step 2, crossover. GA crossover of two chromosomes at gene *j* is calculated as

(10) $$\begin{array}{c} \alpha_{kj}=\alpha_{kj}\left(1-\beta \right)+\alpha_{lj}\beta \\ \alpha_{lj}=\alpha_{lj}\left(1-\beta \right)+\alpha_{kj}\beta \end{array}\}$$

where $\alpha_{kj}$ and $\alpha_{lj}$ are the gene *j* of chromosome *k* and chromosome *l,* respectively, *β* is the cross coefficient, which is a random number between 0 and 1;

- Step 3, evolution. GA evolution of $\alpha_{ij}$ is calculated as

(11) $$\alpha_{ij}=\{\begin{array}{c} \alpha_{ij}+\left(\alpha_{ij}-\alpha_{max}\right)f\left(g\right),\gamma >0.5\\ a_{ij}+\left(\alpha_{min}-\alpha_{ij}\right)f\left(g\right),\gamma \leq 0.5 \end{array}$$

(12) $$f\left(g\right)=\gamma {\left(1-g/G_{max}\right)}^{2}$$

where $\alpha_{max}$ and $\alpha_{min}$ are the upper and lower threshold of $a_{ij}$, respectively, *g* and $G_{max}$ are the current and maximum number of GA evolutions, respectively, *γ* is the evolution coefficient, which is between 0 and 1.

It is necessary to repeat the GA optimization until the maximum fitness is obtained. The individual chromosome code with the maximum fitness is the optimal solution. By decoding the optimal solution, optimal initial input weights $\omega_{ij}$ and hidden layer bias $b_{j}$ can be obtained for the ELM.

#### 3.2.3. IGA Using Levy Flight

In the process of model testing, it was found that fixed cross coefficient *β* in GA crossover can lead to mild crossover and excessive crossover at the later stage, which affected the practical application of GA-ELM. To solve this problem, the Levy flight algorithm is introduced to improve GA algorithm. A Levy flight is a random walk strategy with non-Gaussian distribution. During the walk, the Levy flight is accompanied by frequent short walks and occasional long walks, so it effectively balances the mild crossover and excessive crossover of GA [39,40]. Its update formula is as follows:

(13) $$\beta_{i}^{t+1}=\left|\frac{\beta_{i}^{t}}{\beta_{i}^{t}+\tau ⊕Levy\left(\delta \right)}\right|$$

where $\beta_{i}^{t}$ is the current cross coefficient, τ is the random step size, ⊕ is the dot product, the step length $\delta =\frac{\rho }{\left|\eta \right|}$, where ρ and η have Gaussian distribution, and random dynamic search step $\tau =\mathrm{rand}\cdot \cos\left(\pi -\left|1-\frac{i}{N}\right|\right)$, where *i* is the current number of individual and *N* is the maximum number of individual.

## 4. Cases Study

### 4.1. SCADA Data Description

The data in this study come from Damianshan Wind Farm in Wanyuan City, Sichuan Province, China. The wind farm has a total of 33 1.5-MW WTs, with an annual power of 90.489 million kWh. The SCADA in this wind farm records data of 26 parameters, as shown in Table 1, every 1 min.

The output of the model should not only directly reflect the working state of WT, but also have a great impact on maintenance. Among the various failures of WT, the main bearing failure costs the most [18]. Since the main bearing temperature is closely related to the health of the main bearing, the main bearing temperature is chosen as the output of the model in this paper.

The input should be directly related to main bearing and WT, which are: (a) the production parameters, such as active/reactive power; (b) the parameters which are close to the main bearing temperature, such as gearbox front/rear bearing temperature; (c) the environmental condition. In this study, the input of the model contains eight parameters, namely active power, rotor speed, gearbox front bearing temperature, gearbox rear bearing temperature, nacelle ambient temperature, tower vibration, external temperature and wind speed.

### 4.2. WCC Results

**Figure 6:**
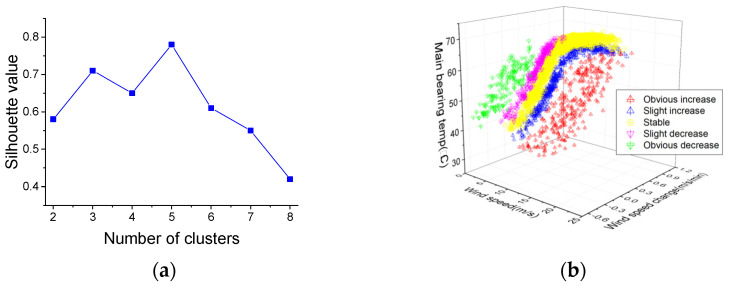
(**a**) Silhouette values of different clusters numbers; (**b**) Three-dimensional visualization of optimal clustering.

The SCADA data from 1 May to 20 May are extracted to partition the wind conditions. In this paper, in order to be more consistent with the wind characteristics, the number of clusters is set from two to eight, and the silhouette value results and the three-dimensional visualization of WCC are shown in Figure 6.

From Figure 6a, it can be seen that when the number of clusters is five, the silhouette value reaches the maximum value of 0.78. This result indicates that it is optimal to segment the wind into five conditions: Condition I (wind speed obvious increase), Condition II (wind speed slight increase), Condition III (wind speed stable), Condition IV (wind speed slight decrease) and Condition V (wind speed obvious decrease). From Figure 6b, five separate wind condition spaces can be clearly observed which also explicitly shows the effects of wind conditions on WT temperature. Table 2 summarizes WCC distribution to further quantitatively understand the clustering results.

As can be seen in Table 2, as far as the wind speed change is concerned, the ranges under the five conditions are different. In terms of wind speed, there is little difference in the ranges under the five conditions. One can see from the results that the wind conditions are clearly partitioned according to this parameter, namely wind speed change, and thus this parameter can be used for subsequent real-time wind condition recognition purposes. It should be noted that wind speed change ranges are open to more than 1.17 m/s/min in Condition I and less than −0.66 m/s/min in Condition V.

### 4.3. Model Validation

#### 4.3.1. WCC Performance Test

**Figure 7:**
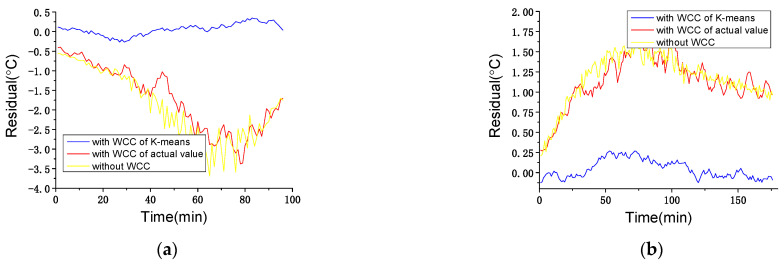
Residual results of Solution I and II in normal working state within wind conditions: (**a**) wind speed increase; (**b**) wind speed decrease.

To verify the effectiveness of WCC using *K*-means clustering, a solution with WCC of wind speed actual value (0–3, 3–12 and >12 m/s) and a solution without WCC are applied as comparison. The algorithms of all three solutions are same as IGA-ELM. Typical data sets of wind speed increase and decrease are chosen as shown in Table 3. It is worth mentioning that, just like the algorithm performance test, the WT works normally in these two periods. The residual results of the three solutions are shown in Figure 7, and the statistical indicators are shown in Table 4.

As shown in Figure 7, the proposed solution with WCC of *K*-means clustering shows much better performance than the other two comparison solutions do, which is also proved by the statistical indicators in Table 4. During the wind speed increase, the residual results’ amplitude of the proposed solution is 0.32 °C, and those of the solution with WCC of actual value and the solution without WCC are 3.49 °C and 3.78 °C, respectively. With WCC of actual value or without WCC, the monitoring (residual) results can be misleading information which can easily trigger false alarms. The same situation can also happen during the wind speed decrease with 0.29 °C vs. 1.56 °C and 1.71 °C. This demonstrates that WCC of *K*-means clustering can improve the accuracy of the model and avoid false alarms.

Additionally, the solution with WCC of actual value and the solution without WCC achieve worse performance during wind speed increase than during decrease. This occurs because the speed of wind speed change can directly determine the delay between the wind speed change and internal temperature change. As mentioned in Section 2, there is a certain delay between wind speed change and internal temperature change, which means wind condition can determine the difference between the predicted value (only based on working state without wind speed change) and the actual value of internal temperature. In these two periods, during the wind speed increase, 54% data (from 9:42 to 10:36) are in Condition I (wind speed obvious increase) and the rest of the data are in Condition II (wind speed slight increase); during the wind speed decrease, only 18.3% data (from 14:58 to 15:31) are in Condition V (wind speed obvious decrease) and the rest of the data are in Condition IV (wind speed slight decrease). The difference of wind condition distribution makes the absolute values of the two comparison solutions’ residual results during the wind speed increase generally larger than that during the decrease, which also reflects the effect of wind condition on WT temperature monitoring.

#### 4.3.2. IGA-ELM Performance Test

To build and test the IGA-ELM model, the learning set and test set are shown as in Table 5. To ensure model accuracy, the learning set should cover the working conditions and state as much as possible only without failures. Similarly, the test set should also contain a variety of working states and wind conditions without failures.

**Figure 8:**
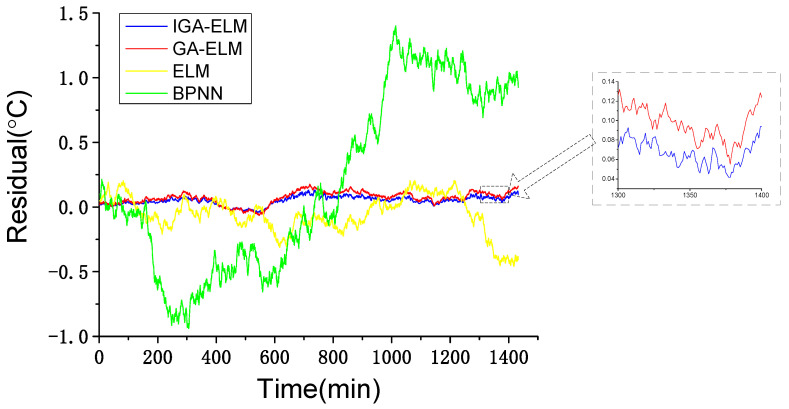
Model testing results of different intelligent algorithms.

In this test, GA-ELM, original ELM and back propagation neural network (BPNN) are used for comparison. The building progress of the comparison models are the same as the proposed model, with WCC. The only difference between the proposed model and comparison models is the intelligent algorithm. The residuals of IGA-ELM, GA-ELM, ELM and BPNN are shown in Figure 8. It should be noticed that the outputs of the models are the residuals of main bearing temperature which is the actual value minus the predictive value. In addition, to quantitatively compare the performance of the testing models, mean square error (MSE = $\frac{1}{s}\sum_{k=1}^{s}{\left(e_{k}\right)}^{2}$), mean absolute error ($\mathrm{MAE}=\frac{1}{s}\sum_{k=1}^{s}\left|e_{k}\right|$) and mean absolute percentage error ($MAPE=\frac{1}{s}\sum_{k=1}^{s}\left|\frac{e_{k}}{Y_{k}}\right|$) are used to analyze the residuals. Statistical indicators of the residuals are shown in Table 6.

As shown in Figure 8, the GA-ELM model and IGA-ELM model have a better performance than the ELM model and BPNN model, demonstrating that the GA optimization is effective. Figure 8 also shows that the residuals of the IGA-ELM model are generally smaller than that of the GA-ELM model. Consistently in Table 6, the IGA-ELM achieves a smaller MSE, MAE and MAPE. This demonstrates that the parameter optimization can improve the accuracy of the model.

### 4.4. Main Bearing Failure Detection

**Figure 9:**
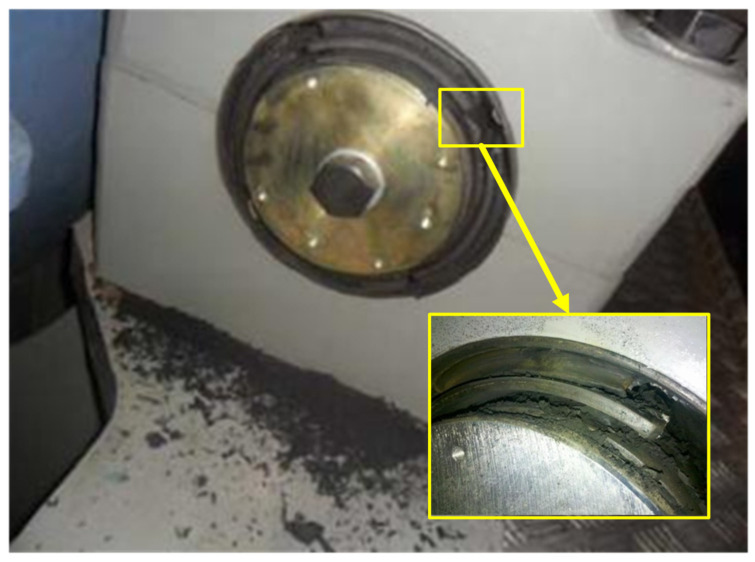
Main bearing offset failure.

**Figure 10:**
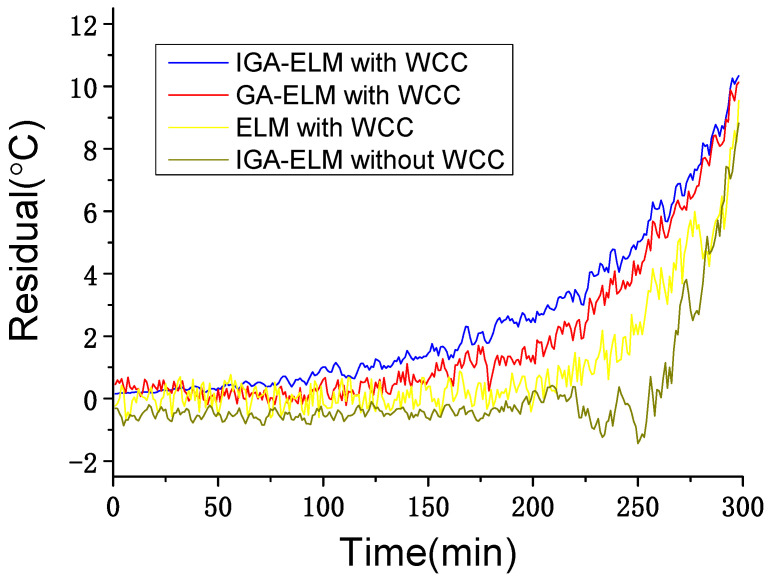
Residual results of different solutions in main bearing offset failure.

To verify the failure detection ability of the proposed solution, a serious main bearing offset occurred in the wind farm, which is as shown in Figure 9, is used as a failure case. The data set of 5 h before the failure happened is shown in Table 7. In this test, solution of IGA-ELM without WCC, solution of GA-ELM with WCC and solution of ELM with WCC are used for comparison. The residual results are shown in Figure 10.

Comparing the two solutions of IGA-ELM in Figure 10, it can be seen that the solution without WCC generally falls behind the solution with WCC by more than 60 min, reaching about 90 min at a residual result of 2 °C. Especially from 9:32 (time point 232) to 10:16 (time point 276), during Condition I (wind speed obvious increase), the solution without WCC shows downward trend over a short time. Additionally, considering the conclusions of the WCC performance test, the solution with WCC exhibits a stable performance during wind condition change, and the residual results are generally less than 0.5 °C. However, for the solution without WCC, due to the wind condition change, residual results under normal working state can be more than 1 °C, sometimes even reaching 4 °C. Thus, the safe range of the solution with WCC is narrower than that of the solution without WCC. If the safe range with WCC is set to ±1 °C and that without WCC is set to ±2 °C, the alarm with WCC can be about 120 min earlier than without WCC. If the safe range with WCC is set to ±0.5 °C and that without WCC is set to ±4 °C, the alarm with WCC can be more than 180 min earlier than without WCC. These results demonstrate that, with WCC, monitoring can achieve earlier failure alarms.

Comparing the three solutions with WCC in Figure 10, for the residual larger than 0.5 °C, the solution of IGA-ELM is averaging 18 and 32 min earlier than that of GA-ELM and ELM, respectively. Especially in the early stages of failure, at a residual of 2 °C, the solution of IGA-ELM is about 37 and 64 min earlier than that of GA-ELM and ELM, respectively; at residual of 1 °C, the solution of IGA-ELM is about 45 and 103 min earlier than that of GA-ELM and ELM, respectively. These results demonstrate that the IGA optimization can reduce the effects of wind conditions on the monitoring and obtain earlier failure alarms.

## 5. Conclusions

WTs are exposed to harsh conditions all year round, and the variability of wind conditions can affect WT monitoring directly. Data show that the main bearing temperature can be more than 5 °C different in the same working state but different wind conditions. Conversely, the data-driven model may fall into a local minimum due to the irregularity of wind condition change and the randomness of initial weights and bias, which can also affect monitoring accuracy.

In this paper, a novel WT monitoring solution based on WCC and IGA-ELM is proposed to solve these problems. On the one hand, considering the effects of wind conditions on the WT temperature monitoring, WCC using *K*-means clustering can partition wind data into several condition clusters, which can make the monitored data more suitable with their corresponding models. On the other hand, ELM is optimized by IGA for the irregularity of wind condition change and the randomness of initial weights and bias, which can improve the accuracy of the model. With testing cases in different wind conditions, it proves that, compared to the solution without WCC or IGA optimization, the proposed solution could reduce false alarms when WT is in a normal working state. A main bearing failure case shows that, with WCC, the alarm can be advanced by at least 60 min. All the cases demonstrate that the proposed solution with WCC and IGA optimization can improve the accuracy of WT temperature monitoring, thereby reducing operation and maintenance costs.

It should be noted that this study focuses on wind speed and its change to describe wind conditions. However, wind conditions also include other factors, such as air humidity and air pressure. In future research, these factors should also be considered to make the WT monitoring more accurate.

## Figures and Tables

**Table 1:** WT parameters in SCADA.

Wind turbine	Gearbox system	Gear oil inlet temp Gear oil sump temp Gearbox front bearing temp Gearbox rear bearing temp Hydraulic pressure Gear oil pressure intake Gear oil pressure pump HSS torque
	Generator system	Rotor speed Main bearing temp Pitch motor 1&2&3 temp
	Converter system	Nacelle ambient temp Hub ambient temp Cooling air temp
	Power system	Active power Reactive power Pitch angle Line voltage Line current Line frequency
	Tower system	Controller cabinet temp Tower vibration
	Environment	External temp Wind Speed

**Table 2:** Summary of WCC distribution.

Distribution	C I	C II	C III	C IV	C V
Wind speed (m/s)	1.76, 18.57	1.04, 18.27	0.08, 24.36	1.35, 14.62	2.59, 11.40
Wind speed change (m/s per min)	0.21, 1.17	0.08, 0.21	−0.05, 0.08	−0.13, −0.05	−0.66, −0.13

**Table 3:** Data sets of wind speed increase and decrease.

Data Set	Start and End Time	Number of Data	Ambient Temperature	Wind Speed
Wind speed increase	12 April 09:00–10:39	100	(13.92, 15.01) °C	(4.64, 15.12) m/s
Wind speed decrease	15 April 14:00–16:59	180	(14.45, 15.89) °C	(3.97, 14.83) m/s

**Table 4:** Statistical indicators of Solution I and II in normal working state during wind speed increase and decrease.

Criteria	Wind Speed Increase			Wind Speed Decrease		
	with WCC of *K*-Means	with WCC of Actual Value	without WCC	with WCC of *K*-Means	with WCC of Actual Value	without WCC
MSE	0.14	2.59	2.85	0.12	0.87	0.95
MAE	0.31	1.98	2.19	0.26	0.83	0.89
MAPE (%)	0.47	3.22	3.48	0.38	1.41	1.54

**Table 5:** Description of the learning and test sets.

Data Set	Start and End Time	Number of Data	Ambient Temperature	Wind Speed
Learning set	1 May 00:00– 20 May 23:59	28,800	(8.41, 31.79) °C	(0.23, 23.62) m/s
Testing set	21 May 00:00– 21 May 23:59	1440	(12.45, 20.02) °C	(4.63, 16.09) m/s

**Table 6:** Statistical indicators of different intelligent algorithms.

Criteria	IGA-ELM	GA-ELM	ELM	BPNN
MSE	0.07	0.10	0.21	0.58
MAE	0.12	0.19	0.59	0.91
MAPE (%)	0.18	0.26	0.73	1.84

**Table 7:** Data sets of main bearing offset failure.

Data Set	Start and End Time	Number of Data	Ambient Temperature	Wind Speed
Failure	18 March 05:40–10:39	300	(−5.58, 0.02) °C	(3.64, 17.86) m/s
